# Effect of a point-of-care ultrasound (POCUS) curriculum on emergency department soft tissue management

**DOI:** 10.1186/s13089-022-00292-4

**Published:** 2022-10-21

**Authors:** Benjamin K. Nti, Whitney Phillips, Elisa Sarmiento, Frances Russell

**Affiliations:** 1 Division of Pediatric Emergency Medicine Division of Clinical Ultrasound Department of Emergency Medicine, Indiana University School of Medicine, 720 Eskenazi Avenue, FT 3, Indianapolis, IN 46202 US; 2 Department of Pediatrics Riley, Hospital for Children at Indiana University Health, Indiana University School of Medicine, 705 Riley Hospital Drive, Indianapolis, IN 46202 US

**Keywords:** Pediatric, Soft tissue, Ultrasound, Training, Curriculum, Emergency department, Point-of-care ultrasound, Infection, Curriculum, Pediatrics, Education

## Abstract

**Background:**

Pediatric emergency department (ED) visits for superficial skin and soft tissue infections (SSTI) have steadily been increasing and point-of-care ultrasound (POCUS) continues to be an effective modality to improve management and shorter ED length of stays (LOS).

**Objective:**

We sought to determine the impact of a soft tissue POCUS curriculum on POCUS utilization, ED LOS, and cost-effectiveness.

**Methods:**

This was a retrospective pre- and post-interventional study of pediatric patients aged 0 to 17 years. Patients presenting to ED with international classification of disease 9 or 10 code for abscess or cellulitis were included. Data were collected a year before and after curriculum implementation with a 1-year washout training period. Training included continuing medical education, greater than 25 quality assured examinations, and a post-test. We compared diagnostic imaging type, ED LOS, and mean charges in patients with SSTI.

**Results:**

We analyzed data on 119 total patients, 38 pre- and 81 post-intervention. We found a significant increase in the total number of POCUS examinations performed pre- to post-curriculum intervention, 26 vs. 59 (*p* = 0.0017). Mean total charges were significantly decreased from $3,762 (± 270) to $2,622 (± 158; *p* = 0.0009). There was a significant trend towards a decrease in average ED LOS 282 (standard error of mean [SEM] ± 19) vs 185 (± 13) minutes (*p* = 0.0001).

**Conclusions:**

Implementation of a soft tissue POCUS curriculum in a pediatric ED was associated with increased POCUS use, decreased LOS, and lower cost. These findings highlight the importance of POCUS education and implementation in the management of pediatric SSTI.

## Introduction

Superficial skin and soft tissue infections (SSTI) including abscesses and cellulitis are common diagnoses in the pediatric population with recent literature showing a steady increase in emergency department (ED) visits [1, 2, 15–17]. The treatment of SSTI frequently depends on the location and depth of infection, and whether there is a fluid collection requiring incision and drainage [1].

POCUS is highly accurate for differentiating abscess from cellulitis and can be used to guide acute management [1]. Its use in the pediatric ED for SSTI is emerging and recent literature has shown that when used with physical examination, POCUS can increase sensitivity and specificity for diagnosing SSTI [1–4]. This includes abscess and cellulitis when compared to physical exam alone. The POCUS application for SSTI has been shown to be favorable to patient outcomes in the acute care setting as it improves diagnostic accuracy for ruling in abscess while reducing invasive intervention that might not be needed [3, 13, 14]. Additionally, children receiving POCUS for SSTI experienced shorter ED length of stays (LOS) when compared to children receiving radiology-performed ultrasound (RUS) [3].

As the practice of pediatric POCUS continues to evolve, the effectiveness of training programs established to support various applications such as SSTI is vital to the success of implementation. Many training curriculums exist, but few assess the impact on patient care and management processes to support the routine use of POCUS for diagnostic evaluations in the pediatric emergency department [4, 6].

We sought to determine the impact of a soft tissue POCUS curriculum on the management of pediatric SSTI by assessing POCUS utilization, ED length of stay, and performing a cost analysis before and after implementation of a structured POCUS curriculum.

## Materials and methods

### Study setting and population

This was a retrospective pre- and post-study conducted 1 year before and after implementation of a POCUS SSTI training curriculum in a pediatric ED. The study took place at an urban academic pediatric ED with over 60,000 annual visits. It was deemed exempt by the Institutional Review Board.

### Study participants

**Fig. 1:**
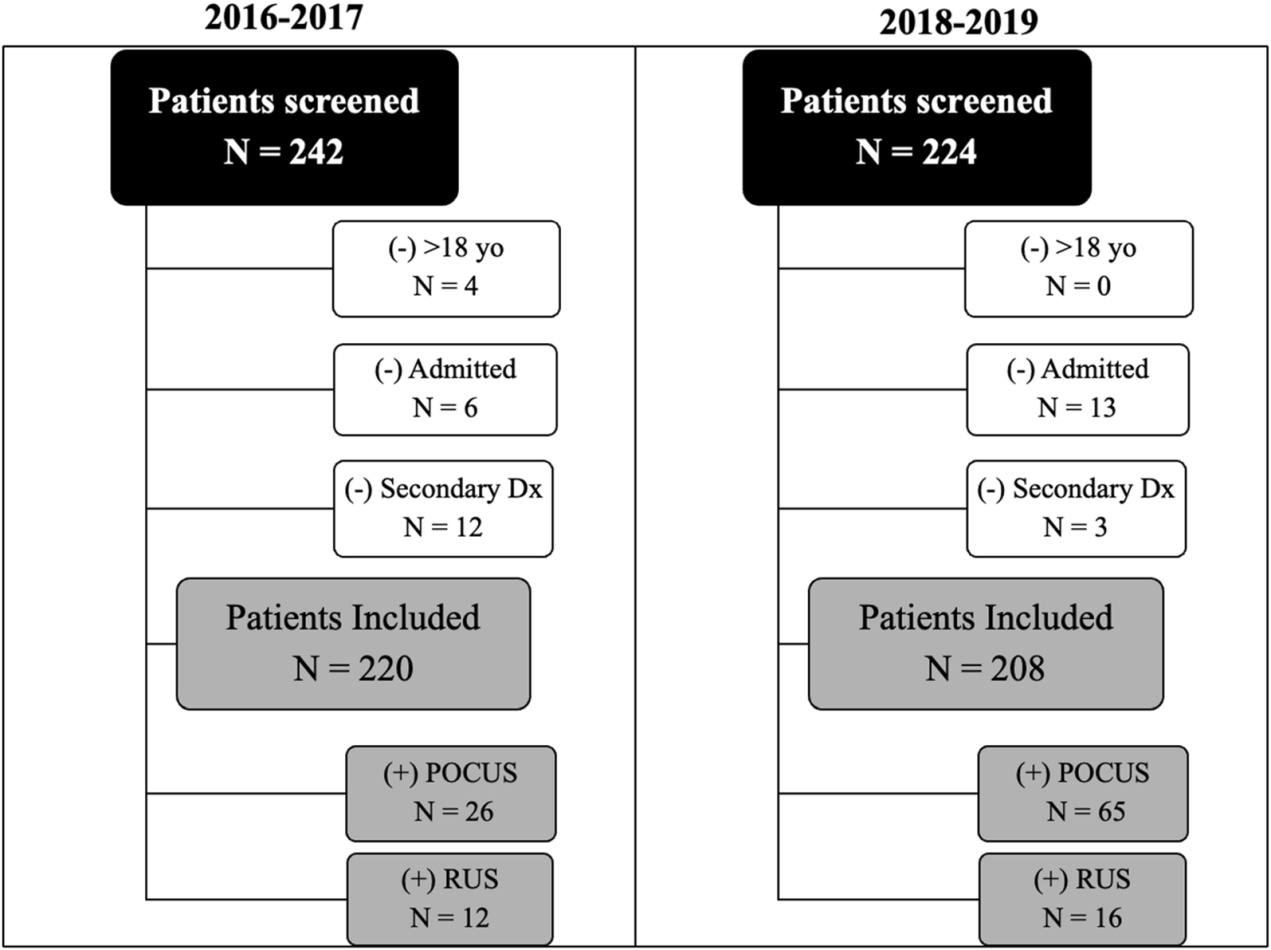
Patient participant flowchart with inclusion and exclusion applied to patient selection pre- and post-intervention of SSTI curriculum

Pediatric patients (0–17 years of age) who presented to the ED from July 1st, 2016, to June 30th, 2017 (pre-implementation) and July 1st, 2018, to June 30th, 2019 (post-implementation) with an SSTI diagnosis met the inclusion criteria for the study. There was a 12-month washout period from July 1st, 2017, to June 30th, 2018, while the training curriculum was implemented. During this washout period, participants were trained and were able to successfully complete the curriculum while routine SSTI care was provided. Pre and post-assessment groups remained the same during the entire length of the study. Patients were included if they had a final diagnosis of SSTI as identified in the electronic medical record by international classification of diseases (ICD) revision codes, 9th (ICD-9 682.2, 682.3, 682.6, 682.8, 682.9, 685.0, 685.1, 686.8, 686.9, 709.8, and 709.9) and 10th (ICD-10 L03.319, L03.119, L03.11, L03.81, L03.818, L05.01, L05.91, L08.9, L99, L03.221, L03.22, L03.317, L03.312). Patients with a secondary diagnosis, complicated infection including those requiring hospital admission and surgical intervention were excluded (Fig. 1). We collected patient demographic information, diagnostic imaging type including POCUS and radiology ultrasound examinations performed, and patient disposition.

### Soft tissue POCUS training

Pediatric emergency medicine faculty and fellows underwent training on soft tissue POCUS to improve knowledge, skill, and comfort to integrate this tool in clinical management of patients. At the time of the study, 14 fellowship-trained pediatric emergency faculty and 8 pediatric emergency fellows took part in training. For the training, faculty were required to complete 1-h didactic sessions, hands-on instruction with supervision by a fellowship-trained POCUS expert, complete 4 h of continuing medical education (CME), successfully pass a competency assessment, and complete 25 quality assured soft tissue POCUS examinations as described previously [3, 5]. Content covered during the didactic sessions included techniques, equipment selection, soft tissue anatomy, image acquisition, differentiating various SSTI and soft tissue edema pathology, differentiating common types of soft tissue foreign body and clinical integration. The hands-on education comprised clinical scans in the emergency department. Faculty obtained CME asynchronously on through institutional and departmental POCUS workshops.

### Sonography and image software

POCUS examinations were completed using a Zonare ZS3 (Mindray, Shenzhen, China) with the linear transducer. Images from the ultrasound system were wirelessly saved to an image archiving and workflow solution (Qpath, Telexy Healthcare, Maple Ridge, BC, Canada) designed to provide immediate feedback and quality assurance.

Radiology ultrasound was conducted in a pediatric radiology department housed adjacent to the main ED with 24-h availability. The ultrasound department is staffed with sonographic technicians who acquire the images and upload them to the institution’s picture archiving and communicating system (PACS) for review and interpretation by a board-certified pediatric radiologist.

### Billing

Using the corresponding CPT codes for SSTI for POCUS and RUS, data concerning charges toward the patients’ payer were collected via the institutions professional billing services and our hospital system’s finance office. Cost was calculated from these charges which included technical and professional component of the radiology services. POCUS rates were set by payer-negotiated reimbursement through the institution’s finance office which included technical and professional fees billed through a third-party billing service.

### Statistical analysis

For continuous measures, we reported descriptive summary statistics such as mean and standard error of the mean. The Wilcoxon test was used to test for differences over time. For categorical measures, we reported frequency and percentages. The Chi-square test was used to determine differences among proportions of ultrasound examination. *P* < 0.05 was considered significant. All statistical analysis was performed using SAS 9.4. The study was powered by a sample size of 91 based on a confidence level of 95% and an alpha of 5% assuming a target population 100 which was based on approximately 50% of actual population of estimated patients with SSTI.

## Results

A total of 242 patients were identified based on ICD-9 and ICD-10 codes for abscess or cellulitis in the pre-intervention cohort, with 220 patients meeting inclusion criteria. Thirty-eight out of 220 (17.3%) received ultrasound imaging as part of their ED workup, 26 out of 38 (68.4%) received POCUS, 12 (31.6%) received radiology-performed US, and 11 (28.9%) received both. Post-intervention, 224 patients were screened with 208 patients meeting inclusion criteria. Fifty-five out of 208 (26.4%) received ultrasound imaging as part of their ED workup, 39 out of 55 (71.0%) received POCUS, 16 (29.1%) received radiology-performed US, and 6 (10.9%) received both (Fig. 1).

There was no significant difference from pre- to post-intervention groups when comparing age and race. The average age of patients with ultrasound imaging performed was 7.92 (standard error of mean [SEM] ± 0.99) years of age in the pre-intervention cohort and 6.83 (± 0.65) in the post-intervention cohort. Most study participants were Caucasian with 71% in the pre-intervention cohort and 58% in the post cohort (Table 1). The number of female patients included significantly increased from the pre- to post-intervention groups, 42% vs 67% (*p* = 0.0075). Cellulitis was the predominant discharge diagnosis both pre- and post-intervention with 69.4% and 73.4% patients for cellulitis and 26.5% and 20.2% patients, respectively, for abscess overall (Table 1). The majority of infections were located in the lower extremities (Table 1).

**Table 1:** Demographics and characteristics of SSTI between pre- and post-intervention patients.

Demographics			
	2016–2017 *N* = 38	2018–2019 *N* = 81	*P-value*
Age in years (± SEM)	7.92 (± 0.99)	6.83 (± 0.65)	0.3252
Gender (%)			0.0075
Female	16 (42.11)	55 (67.90)	
Male	22 (57.89)	26 (32.10)	
Race (%)			0.1873
Asian	0 (0.0)	1 (1.23)	
Black	10 (26.32)	33 (40.74)	
White	27 (71.05)	47 (58.02)	
Unknown	1 (2.63)	0 (0.0)	
SSTI infection			0.1378
Cellulitis	26 (69.4)	60 (73.4)	
Abscess	10 (26.5)	17 (20.2)	
Other	2 (5.31)	4 (4.94)	
SSTI location (%)			0.1727
Upper extremity	6 (15.7)	13 (16.0)	
Lower extremity	18 (47.4)	36 (44.4)	
Buttock	9 (23.7)	11 (13.6)	
Cyst	3 (7.9)	11 (13.6)	
Other	2 (5.3)	10 (12.3)	

*P* = < 0.05 denotes statistical significance

*Min* minimum, *Max* maximum

**Fig. 2:**
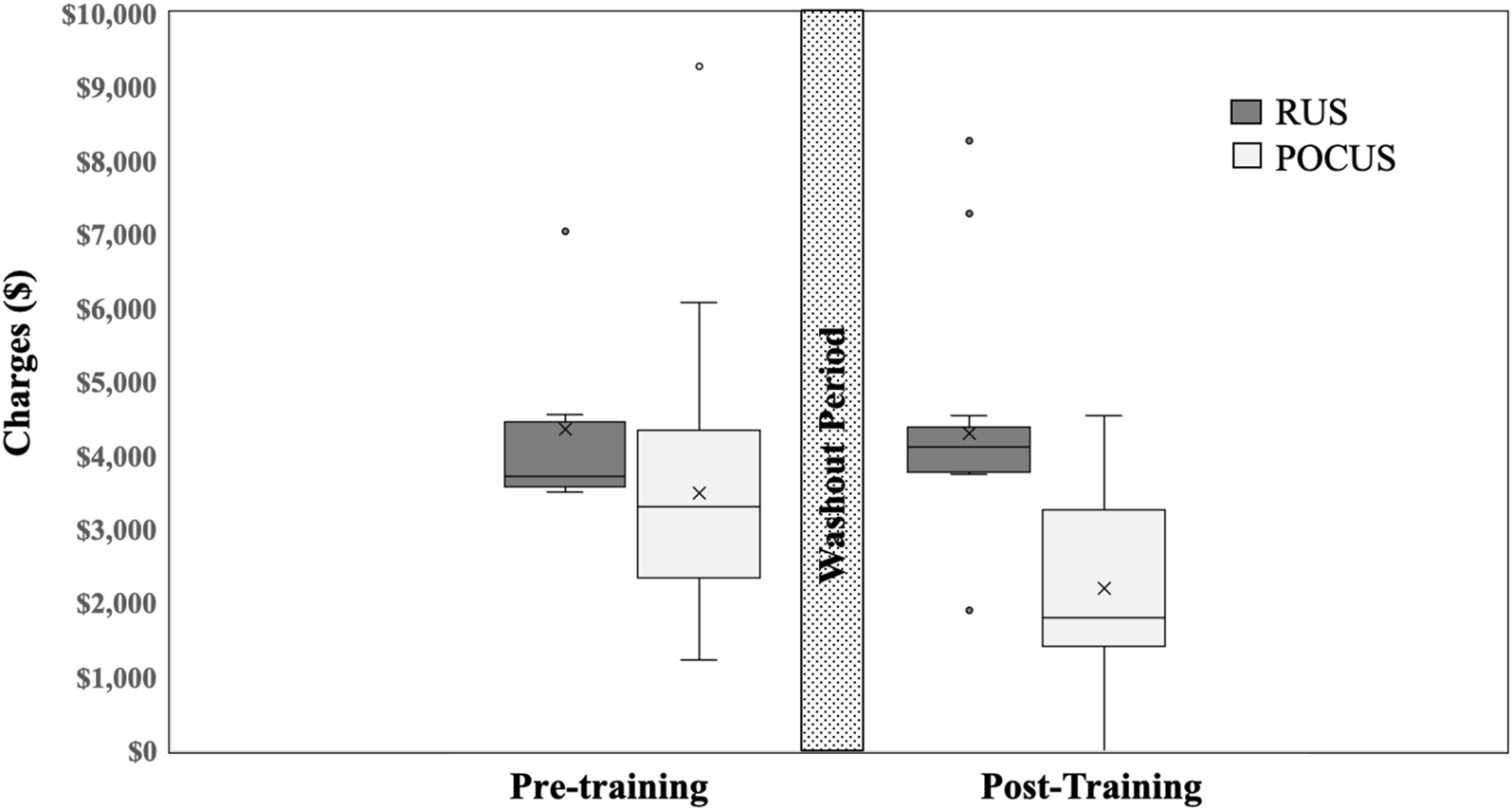
Box plot of ED charges pre- and post-intervention between POCUS and radiology ultrasound (RUS) with 1-year washout period (gray bar). Circular symbol = outliers; X = mean

Total mean charges decreased significantly from pre- to post-intervention, $3,762 (± 270) vs. $2,622 (± 158; *p* = 0.009; Table 2). The mean charge for imaging was significantly lower for patients with POCUS after training than for patients with a RUS performed in the pre- and post-intervention cohorts ($3,491 (± 345) vs $2,193 (± 116), *p* = < 0.0001 and $4,349 (± 376) vs $4,291 (± 175),* p* = 0.1411, respectively); see Fig. 2.

**Table 2:** Clinical experience. Description of imaging types, ED length of stay, and charges for corresponding imaging

Imaging (*N* = 119)			
Imaging type	2016–2017	2018–2019	*P-value*
Total ultrasound (%)	38 (17.3)	81 (38.9)	0.00001
POCUS alone (%)	26 (68.4)	59 (72.8)	0.6188
POCUS + RUS (%)	0 (0.0)	6 (7.4)	0.3714
POCUS + CT (%)	1 (2.6)	0 (0.0)	0.6286
RUS alone (%)	10 (26.3)	16 (19.7)	0.9305
RUS + CT (%)	1 (2.6)	0 (0.0)	0.6286
Mean ED charges, $	3,762 (± 270)	2,622 (± 158)	0.0009
POCUS charge (± SEM)	3,491 (± 345)	2,193 (± 116)	0.0001
RUS charge (± SEM)	4,349 (± 376)	4,291(± 175)	0.1411
Mean ED LOS, minutes	282 (± 19)	185 (± 13)	0.0001
POCUS LOS (± SEM)	161 (± 13)	140 (± 10)	0.0001
RUS LOS (± SEM)	266 (± 28)	239 (± 21)	0.0001

*P* < 0.05 denotes statistical significance

*Min* minimum, *Max* maximum

**Fig. 3:**
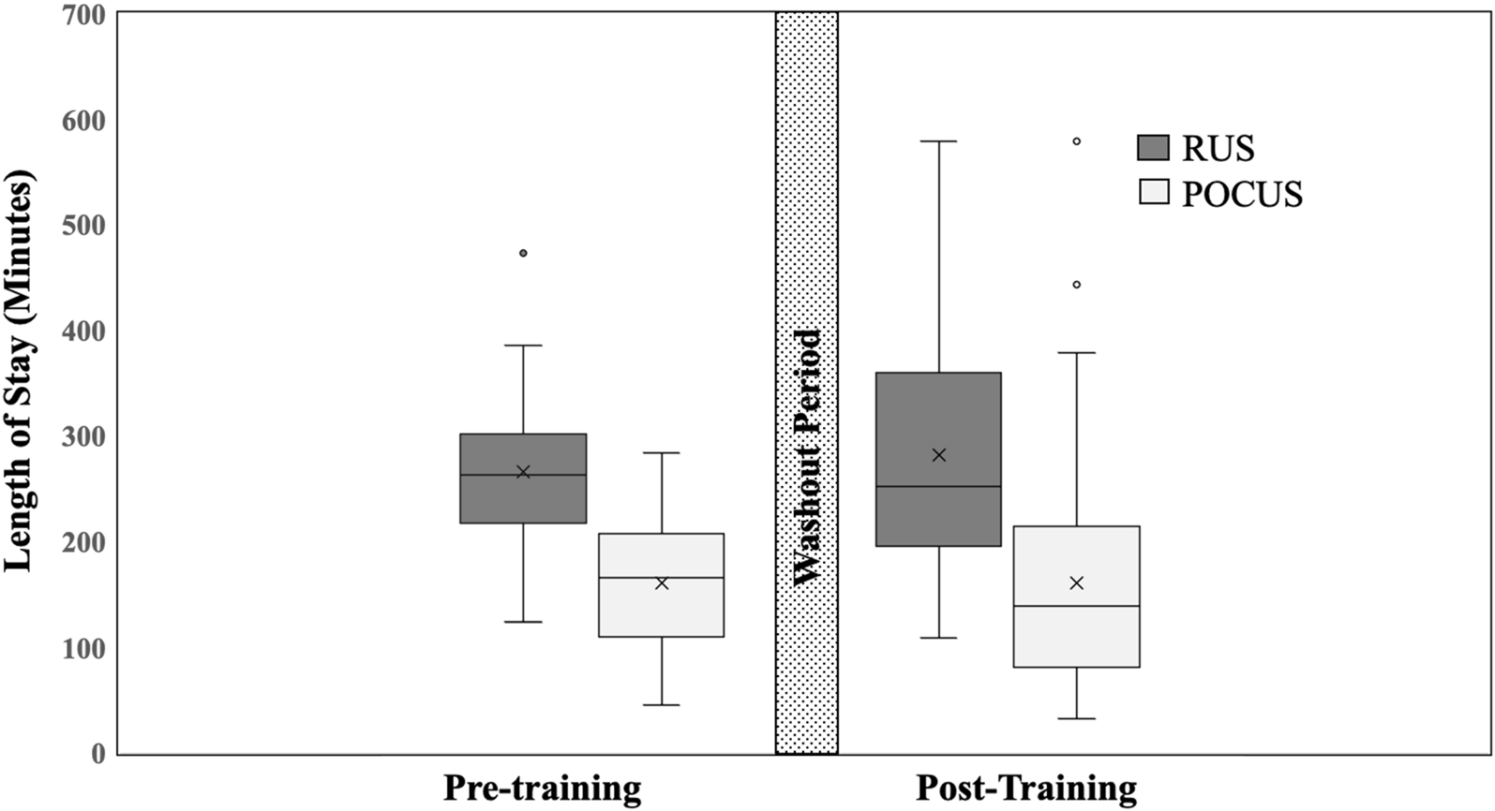
Box plot of ED length of stay (LOS) in pre- and post-intervention between POCUS and radiology ultrasound (RUS) with 1-year washout period (gray bar). Circular symbol = outliers; X = mean

From pre- to post-intervention, the rate of patients receiving ultrasound increased, 17.3% vs 38.9% (*p* = 0.00001) and the frequency of patients receiving POCUS increased, 26 (68.4%) vs 59 (72.8%, *p* = 0.6188). Overall men ED LOS of significantly decreased from 282 (± 19) minutes pre-intervention to 185 (± 13) minutes post-intervention, *p* = 0.0001 (Table 2). The patients receiving POCUS in the pre- and post-intervention cohorts had significantly shorter LOS when compared to patients with radiology ultrasound (161 (± 13) vs. 140 min, *p* = < 0.0001, and 266 (± 28) vs. 239 (± 21), *p* = < 0.0001, respectively) (Table 2, Fig. 3).

## Discussion

POCUS is becoming more widely used in the pediatric ED to guide acute treatment decisions in patients with SSTI [3, 4]. Little is known about the clinical impact of POCUS training curriculums on patient management. Previous studies show that the use of POCUS can augment the clinical evaluation for management of ED patient with SSTI [3, 19]. In this novel study, we show the clinical impact of a soft tissue POCUS curriculum on pediatric ED patients with a presenting SSTI which can influence provider use of POCUS, improve ED metrics, and decrease cost burden. As a result, we found that a streamlined curriculum for faculty and fellows was associated with an increased frequency of POCUS being performed, significantly lower total mean charges, and a trend towards shorter ED LOS.

Unsurprisingly, we found the rate of POCUS examinations being performed was higher in the post-intervention group, (65/208) 31.2% from 26/220 (11.8%), *p* = 0.6188. This is consistent with prior research showing that SSTI training programs can enhance technical ability and POCUS utilization in the emergency department [1, 8]. Interestingly, a few of the patients (7.5%) in the post-intervention cohort received both POCUS and RUS evaluation. This is important because while most of the faculty and fellows were comfortable acquiring and interpreting the images, a few indeterminate scans may have required further evaluation and impression by the radiologist. The use of CT for further evaluation of complex SSTI is common practice [15]. While 2 patients required additional CT in the pre-intervention, there were no CT scans utilized following the training. It is possible that this was associated with the implementation of the curriculum, but more likely due to the exclusion of patients with complex SSTI. Nevertheless, the practice of utilizing CT scans for non-complex SSTI was non-existent after the intervention. Further review of patient charts did not show any complications as a result of clinical management of SSTI in our patient cohort.

Evidence suggests that the use of ultrasound for bedside clinical evaluation is cost-effective [10–12, 18]. Common applications specific to the emergency setting such as the FAST not only decrease reliance on imaging such as the computed tomography (CT) for evaluation in stable patients, but also promote an efficient cost-effective approach to patient care [9]. Similarly, we found significantly lower total mean charges from pre- to post-intervention, $3,762 vs. $2,622, *p* = 0.009. In this cost-effective analysis, the difference in cost when ultrasound alone was used compared to when radiology ultrasound was estimated at $1,100. As POCUS continues to emerge, the cost-effectiveness is likely to be variable from institution to institution and may also vary at various time points due to internal fee adjustments based on factors such as professional and technical fees. We found no cost variation during the time period of the retrospective data analysis. Similarly, to prior POCUS studies, our data suggest that although it may be limited, the inclusion of POCUS promotes cost-effective care concomitantly with ED efficiency [10].

It is well documented that SSTI can be made clinically without POCUS, but its use can augment patient care in the acute care setting [1–3]. Our retrospective cohort sample was significantly smaller than other studies due to stringent inclusion criteria applied to the data collection and because lower frequency of ultrasound use for evaluation of SSTI at our instruction. Another primary benefit of POCUS is the potential to enhance ED patient experience metrics. This is most evident in evaluation of SSTI as documented in prior literature where SSTI evaluation by POCUS decreased LOS when compared to radiology-performed ultrasound [11]. Similarly, to the study by Lin et al., our study showed a trend toward a significant decrease in ED LOS, 282 min pre-intervention vs. 185 min post-intervention, *p* = 0.001. However, while this study adjusted findings for relevant clinical variables, it included potential confounding diagnoses that may affect LOS and cost-effectiveness. Furthermore, it is possible that factors such as triage and registration, peak times, and staffing may have had minimal effect on the LOS time collected.

## Limitations

There are several limitations to consider in this study. This study is based on a one-group pre- and post-assessment after an intervention which lacks a control group for comparison. Additionally, this type of study may have confounding factors that might not be associated with the intervention which may affect the validity of the study. The majority of our patients were Caucasian, which raises concerns about whether this can be generalizable. Additionally, the lack of external validity in single-center studies may limit the true effect of soft tissue POCUS training. Of importance, the smaller sample size which excluded a substantial portion of the patient population may also underestimate the true effect of the training curriculum. However, evidence does support the beneficial impact of soft tissue training to enhance management of SSTI in the pediatric ED [3]. Soft tissue POCUS required that a trained faculty or fellow had to be present and be confident enough in their skill and interpretation to apply US findings clinically while avoiding complete radiology ultrasound evaluation. However, even though the majority of our physicians were credentialed during this study, not all were confident enough to use the study frequently, but this is likely to improve as we gain more understanding into the longitudinal effects of POCUS implementation curriculums on clinical outcomes and patient experience.

## Conclusions

After training pediatric emergency medicine faculty and fellows to use POCUS for SSTI, clinical utilization of this application increased while being cost-effective and decreasing the LOS for patients presenting to ED with SSTI. This suggests that investing in POCUS training for applications such as SSTI can enhance the patient experience while keeping costs low and avoiding unnecessary invasive procedures.

## Summary

Soft tissue evaluation is a high-yield POCUS application that can augment patient care while improving management in the pediatric acute care setting. This study emphasizes the importance of establishing a curriculum to enhance its use by clinicians. To this regard, our findings showed an improved utilization with an associated impact on efficiency of patient care and cost-effectiveness.

## Meetings

American Academy of Pediatrics Virtual National Conference and Exhibition 2020.
